# Sleep Genetics and Cognitive Changes over Time: The Moderating Effect of Age and the Role of Brain

**DOI:** 10.3390/genes16010021

**Published:** 2024-12-26

**Authors:** Angeliki Tsapanou, Seonjoo Lee, Silvia Chapman, Niki Mourtzi, Christian Habeck, Yaakov Stern

**Affiliations:** 1 Cognitive Neuroscience Division, Department of Neurology, Columbia University, New York, NY 10032, USA; sc4056@cumc.columbia.edu (S.C.); ch629@cumc.columbia.edu (C.H.); ys11@columbia.edu (Y.S.); 2 Department of Psychiatry, Columbia University, New York, NY 10032, USA; sl3670@cumc.columbia.edu; 3 Department of Neurology, National and Kapodistrian University of Greece, 11528 Athens, Greece; nikimourtzi23@gmail.com

**Keywords:** sleep, polygenic index, cognitive changes, brain morphometry, aging

## Abstract

Background: Sleep plays a crucial role in cognitive performance and cognitive changes in aging. In the current study, we investigated the role of sleep duration genetics in cognitive changes over time and the moderating effect of age. Methods: Participants were drawn from the Reference Abilities Neural Network and the Cognitive Reserve studies of Columbia University. Each participant underwent an evaluation of sleep function and an extensive neuropsychological assessment. Published GWAS summary statistics from a polygenic score for sleep duration (Sleep PGI) were used to derive Sleep PGI in our study. We examined whether this Sleep PGI is associated with cognitive changes over a 5-year follow-up and if age moderates this effect. Analysis was performed after first being adjusted for age group (young: 20–44; middle: 45–64; old: 65–80), sex, education, the first four principal components, intracranial volume (ICV), mean cortical thickness, and total gray matter volume. We included ICV, mean thickness, and total gray matter volumes as time-varying covariates. We further included interactions of time with age and the first four PCs. Results: A total of 96 white-only participants were included, aged 24 to 78 years old. In the fully adjusted model, age-specific analysis showed that in younger individuals, higher Sleep PGI was associated with lower rates of cognitive decline in speed of processing. Conclusion: Genetic variants associated with sleep duration significantly influence performance in speed of processing, with age playing a critical moderating role, over and above brain morphometry. A genetic predisposition for longer sleep duration can work as a protective factor against decline in the speed of processing in young adults.

## 1. Introduction

Sleep dysfunction is associated with cognitive deficits, cognitive changes over time, and incident dementia [1,2]. More specifically, excessive daytime sleepiness is often considered an early sign of cognitive decline in cognitively healthy older adults [3]. An inverted U-shaped association between nighttime sleep duration and cognitive decline is also reported, indicating that cognitive function should be monitored in individuals with insufficient or excessive night sleep duration [4]. In both younger and older adults, seven hours of sleep per day is linked to the highest level of cognitive performance, with performance decreasing for every hour below and above this sleep duration [5]. Individuals who sleep between six and eight hours have greater gray matter volume in specific brain regions [5], indicating the significant association between the modifiable factor of sleep duration and cognition in the bigger picture of structural brain health.

Ever since it was recognized that sleep is genetically regulated, research has begun to focus on the genetics behind sleep disorders and the molecular mechanisms of sleep [6]. Genetic studies have identified numerous loci associated with sleep traits such as duration, quality, and timing, highlighting the complex and polygenic nature of sleep regulation [7]. These genetic substrates not only influence fundamental sleep architecture but also play a role in susceptibility to sleep disorders such as insomnia and circadian rhythm disruptions. The focus on genetic substrates of sleep is intended to isolate the influence of genetic predispositions on cognitive outcomes, independent of actual sleep performance [8]. This approach is not meant to directly measure sleep function but rather to explore how inherent genetic factors, which contribute to sleep regulation, might independently influence cognition over time. By isolating the genetic components of sleep, we aim to uncover mechanisms that predispose individuals to cognitive changes, regardless of observed sleep behaviors. This allows for a deeper understanding of the interplay between genetic predispositions, sleep, and cognition—insights that may be overlooked if genetic factors are treated merely as covariates or secondary variables.

In a cross-sectional study, our group demonstrated that the sleep duration polygenic index (Sleep PGI) was positively associated with cognitive performance [9]. Specifically, a genetic predisposition for a longer sleep duration was correlated with better outcomes in fluid reasoning, processing speed, and language abilities. This association was observed across the adult age range, although it was primarily driven by younger adults. Furthermore, in a separate longitudinal cohort of older adults without dementia, we identified links between Sleep PGI and differential rates of cognitive decline over time. These findings suggest that common genetic variants influencing sleep duration may play a significant role in shaping cognitive health and supporting healthy aging [10]. 

These studies support the existence of a relationship between sleep duration and cognitive performance, with evidence showing that both short and long sleep durations can impact cognitive functions such as memory, attention, and processing speed [11], and that genetic factors play a crucial role in determining individual sleep duration. Therefore, in the current study, we examined the association between Sleep PGI and 5-year cognitive change and assessed whether this association differs with age. We included the potential moderating role of age in this association, and controlled for change in brain morphometry. In a secondary analysis, we addressed the issue of the impact of age more directly by examining interactions between other variables and age group (young, middle, and old).

## 2. Methods

Participants: Participants were drawn from the Reference Ability Neural Network (RANN) and the Cognitive Reserve (CR) studies. The RANN study was designed to identify networks of brain activity associated with performance across adulthood for each of the four following reference abilities: memory, fluid reasoning, the speed of processing, and language [12]. The CR study was designed to elucidate the neural underpinnings of the cognitive reserve and the concept of the brain reserve [13]. All participants were non-Hispanic white native English speakers, were right-handed, and had at least a fourth-grade reading level. Study participants were also required to be free from any major neurological or psychiatric conditions that could affect their cognition. Careful screening excluded participants with MCI or AD. Additional inclusion criteria for participants required a (1) score equal to or greater than 130 on the Mattis Dementia Rating Scale [14] to guarantee a cognitively normal status; (2) minimal or no functional capacity complaints [15] and; (3) complete data on imputed genome-wide genotyped sleep (GWAS), cognitive performance in all domains, and socio-demographic variables (sex, age, and education). Participants were evaluated at baseline and at a 5-year follow-up. Both RANN and CR studies are approved by the Institutional Review Board of Columbia University. More detailed information about the two studies can be found in previous publications [12,16,17,18,19].

Genome-wide SNP-genotyping: Each participant had venous blood drawn during their visit to Columbia University. DNA samples were obtained via whole-blood extraction. Genotyping was performed using Omni 1M chips, and we worked according to Illumina procedures. Genotype calling was performed using GenomeStudio v.1.0. Quality control was applied to both DNA samples and SNPs. Specifically, samples were removed from further analysis if they had call rates below 95%, sex discrepancies, or relatedness.

GWAS imputation: GWAS data for all study participants were imputed using the Haplotype Reference Consortium (HRC v1.1) panel through the Michigan Imputation online server [20]. The HRC is a reference panel of 64,976 human haplotypes involving 39,235,157 SNPs. It was constructed using whole-genome sequence data from 20 studies involving people of predominantly European ancestry [21].

Polygenic index (PGI): To establish the Sleep PGI, we used the summary statistics from a GWAS of sleep duration conducted by Dashti et al. [22] that included 446,118 adults of European ancestry. PGI scoring was performed using PRSice software v2.3.5 [22], following the clumping and thresholding (C+T) approach, as originally described by the International Schizophrenia Consortium. We included all SNPs, regardless of *p*-value. To ensure that only independent markers were included in the computed PGI, we conducted linkage disequilibrium clumping using an R2 threshold of 0.1 and a 250 kb sliding window. Markers within the major histocompatibility complex (MHC) LD region of chromosome 6 (chr6:27–33Mb, hg19) were also excluded from the PGI due to the presence of complex patterns of long-range linkage disequilibrium within this region. For each remaining SNP, we computed the weighted count of cognition-associated alleles (0, 1, or 2), with the weights determined by the coefficient estimated in the GWAS. We then computed the average weighted count across all SNPs to yield the PGI. PGI computation was performed using the PRSice software v2.3.5 [22]. PGI values were z-transformed for the current analysis.

Principal Components: to account for population structure, we computed the Principal Components (PCs) of the whole sample using Plink software v1.9 [23], and we used the first four PCs as covariates in our analysis.

Brain structure undergoes changes with aging, and cortical thickness and gray matter volume are correlated with cognitive performance and sleep [24]. It is therefore important that these structural brain measures are taken into account when investigating the relationship between genetic predispositions, sleep duration, and cognitive performance. Intracranial volume (ICV), mean cortical thickness, and total gray matter volume: A T1-weighted Magnetization-Prepared Rapid Acquisition Gradient Echo (MPRAGE) scan was performed with Echo Time/Repetition Time (TE/TR) values of 3/6.5 ms and a flip angle of 8°, an in-plane resolution of 256 × 256, a field of view of 25.6 × 25.6 cm^2^, and 165–180 slices in the axial direction with a slice thickness/gap of 1/0 mm. FreeSurfer (v5.1.0) software, designed for human brain imaging analysis (http://surfer.nmr.mgh.harvard.edu/ (accessed on 15 June 2017)), was used for the reconstruction of the T1 scans [25,26]. 

Neuropsychological evaluation: Each participant underwent an extensive neuropsychological evaluation. From the neuropsychological battery, we derived four cognitive domains: memory, fluid reasoning, speed of processing, and language. The following tests were used: for memory: Selective Reminding Test (SRT) [27], Long-Term Storage, Consistent Long Term Retrieval, Total words recalled on the last trial. For fluid reasoning: Wechsler Adult Intelligence Scale (WAIS-III) matrix reasoning raw score, WASI-III letter-number sequencing raw score, and Block design test, total correct [28]. For speed of processing: WAIS-III digit-symbol total correct, Trail Making Test (TMT-A) total time [29], and Stroop Word Raw Score [30]. For language: WAIS-III language test, the Wechsler Test of Adult Reading (WTAR) [31], and the American National Adult Reading Test (AMNART), errors [32]. Z-scores were computed for each cognitive task, and each participant, based on the means and standard deviations (SD) of all participants. Scores for TMT-A time and AMNART errors were transformed so that higher score indicates better performance, following the directionality of the other tests.

Diagnosis: the diagnosis of the clinical/cognitive status of each participant was achieved through diagnostic consensus meetings of all the researchers and main investigators, both neurologists and neuropsychologists.

## 3. Statistical Analysis

Descriptive statistics of the demographic characteristics and baseline cognitive performance measures were presented by age group using the mean value, with standard deviation (SD) for continuous variables and frequencies with percentages for categorical variables. We tested the two following hypotheses:

**Hypothesis 1 (H1).**  *Sleep PGI is associated with cognitive change over time in the different cognitive domains.*

**Hypothesis 1 (H2).**  *Age moderates the effect of Sleep PGI on cognitive change*.

Analysis was performed after first adjusting for age group (young, middle, and old), sex, education, and the first four PCs of the SNP data. Intracranial volume (ICV), mean cortical thickness, and total gray matter volume were included as time-varying covariates. As independent variables, we further included the interactions of time with age and the first four PCs.

For H1, we used a linear mixed-effect model, using reference abilities as the dependent variable. We employed time (baseline vs. follow-up), Sleep PGI, and their interactions, as well as covariates, as fixed effects. Random intercepts of subjects were included to account for within-subject correlation occurring due to repeated measurements. The PGI x time interaction term was tested using an F-test and the degree of freedom was estimated via Satterthwaite’s method.

For H2, we included age group (young, middle, and old) and 2-way and 3-way interactions with time and Sleep PGI as fixed effects. If the 3-way interaction was significant, we further performed contrast analysis to quantify the effect of PGI on changes in cognition by age groups. The PGI x time x age group interaction terms were jointly tested using an *F*-test. For the significant 3-way interactions, we performed a post hoc contrast analysis to quantify the effect of Sleep PGI on cognitive changes by age group. 

We performed multiple comparisons correction to control for the false discovery rate [33] within each hypothesis. We did not perform multiple comparisons correction for the post hoc contrast analyses.

Further, as a sensitivity analysis, we repeated the same analyses with different sets of imaging measures (e.g., thickness only, total gray matter volume only) to assess the robustness of the results.

## 4. Results

A total of 96 participants were included in this study, with an age-range of 24 to 78 years old. The mean education was 16.5 (SD:2.3) years, and there was an almost equal distribution regarding sex (females 49%) (see Table 1).

For Hypothesis 1, across age, Sleep PGI was not associated with cognitive change over time for any of the cognitive domains, even after adjusting for age, sex, education, and PCs (p’s > 0.14). After including the imaging measures (mean cortical thickness and total gray matter volume) in the analysis, the results did not change (Supplementary Table S1, p’s > 0.18).

**Figure 1:**
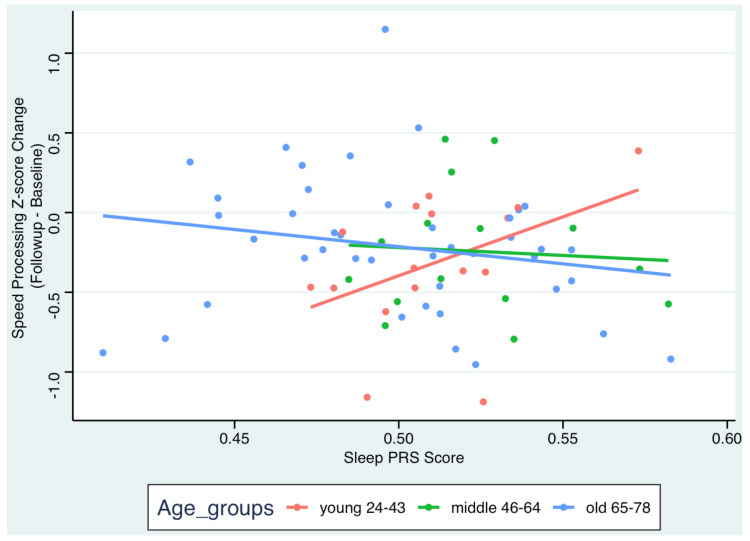
Scatterplot for the association between Sleep PGI and changes in speed of processing by age group. Adjustments for sex, education, 4 PCs, time x 4PCs, ICV, total cortical thickness, and total gray matter volume.

For Hypothesis 2, examining the association between Sleep PGI and cognitive change over time with age as the moderator, even adjusting for age, sex, education, and PCs, the results were also not significant (p’s > 0.11). However, when controlling for brain measures (mean cortical thickness, total gray matter volume) as covariates, results showed that age moderated the association between Sleep PGI and changes in speed of processing (time x Sleep PGI x age group β = −10.24, 95% CI: −18.4–−2.03, *p* = 0.015; see Figure 1, Table 2). The results indicated that a longer Sleep PGI was associated with a better performance in terms of speed of processing. Results for the other cognitive domains were not statistically significant. 

Further, we performed a post hoc contrast analysis to estimate the effect of Sleep PGI on cognitive change by age group (Figure 1, Supplementary Table S3). Lower Sleep PGI was only associated with more cognitive decline regarding the speed of processing among the young group (β = 8.41, 95% CI: 0.52–16.29, *p* = 0.04). This indicates that longer Sleep PGI was associated with lower rates of decline in the speed of processing in young adults. We repeated the same analyses while only controlling for cortical thickness or gray matter volumes. The direction and magnitudes of the estimates remained similar. Results for the other cognitive domains and the other age groups were not statistically significant.

## 5. Discussion

The results of this study offer intriguing insights into the complex interplay between the genetic predispositions of sleep, cognitive performance, and brain structure across different age groups. Although the overall sample did not reveal a significant association between Sleep PGI and changes in performance, further analysis uncovered important age-specific moderating effects on the association between Sleep PGI and cognition over time when controlling for demographic and brain measures. A higher Sleep PGI was associated with lower rates of decline in the speed of processing in young adults. The lack of a significant association in the total sample underscores the importance of considering age-specific factors when investigating the relationships between genetic predispositions, brain structure, and cognitive outcomes. 

In younger individuals, a higher Sleep PGI, which suggests a genetic predisposition for longer sleep duration, was associated with a smaller decline in processing speed. In contrast, older adults may not exhibit the same level of association between Sleep PGI and cognitive decline. It is well known that cognitive decline is caused by a complex interaction between genetic, environmental, lifestyle, and epigenetic factors, but the contribution of each factor on cognitive decline has been rarely examined across different age groups [34,35,36]. One explanation could be that as we become older, the contribution of genetics to cognitive decline becomes less important and other factors such as the co-existence of comorbidities or poor diet take precedence in determining an individual’s susceptibility. Furthermore, it has been suggested that older individuals show less neural plasticity than younger adults [37]. Thus, the neural plasticity reduction in older adults may exert a higher degree of influence on cognitive decline than genetics compared to younger adults.

Sleep PGI was associated with the speed of processing in the model that included two brain measures as covariates: total cortical thickness and total gray matter volume. By contrast, the results of the model that was only adjusted for age, sex, education, ICV, and 4 PCs did not reach the level of statistical significance. This suggests that structural brain metrics are crucial covariates of the relationship between Sleep PGI and cognitive performance. Cortical thickness and gray matter volume are known to decrease with age [38] and are linked to various cognitive deficits [39]. Understanding the interplay between genetics, sleep, and brain structure is essential for creating effective interventions to preserve cognitive function across the human lifespan.

This study expands on our previously reported results where Sleep PGI was significantly associated with cognition cross-sectionally, revealing that genetic predisposition for longer sleep duration was linked to better cognitive performance, particularly among younger adults [9]. By incorporating the moderating effect of age and the inclusion of total cortical thickness and gray matter volume, we now demonstrate that greater Sleep PGI is a protective factor in terms of the speed of processing decline in younger individuals. The absence of a similar moderating effect in older adults could mean that age-related neurodegenerative changes, such as cortical thinning and gray matter reduction, diminish the impact of genetic predispositions on cognitive performance. Additionally, older adults may experience a range of other age-related changes, such as vascular health decline and the increased prevalence of sleep disorders [40], which could influence the moderating effects observed in younger adults. Environmental factors, such as lifestyle, sleep hygiene, and exposure to stress, can significantly influence the relationship between genetic predispositions for sleep duration and cognitive performance [41,42]. 

Age-related cognitive changes can be influenced by brain maintenance, which denotes the relative stability of neural resources and the absence of neuropathologic changes over time, and cognitive reserve, which includes brain processes that enable better-than-expected behavioral performance despite the brain changes associated with aging [43]. Our findings align with the notion that genetic predispositions, particularly those influencing sleep duration, play a crucial role in cognitive health. These findings reinforce the idea that both genetic and environmental factors, such as sleep hygiene and lifestyle, are vital for preserving cognitive health and mitigating the adverse effects of aging on brain structure and function.

The findings from our Sleep PGI–cognitive changes study align with the broader understanding of how sleep and genetics interact to influence cognitive trajectories in aging [44,45,46]. As neuroplasticity diminishes with age, the brain’s ability to adapt and compensate for structural and functional declines becomes increasingly reliant on external and internal factors, including sleep [47,48]. Genetic predispositions, such as those captured by the Sleep PGI, may play a pivotal role in shaping sleep patterns and their downstream effects on cognition. 

There are significant strengths that should be noted. The longitudinal design of the study provides better insight into causal inferences about the relationship between the Sleep PGI and cognition over time. Our approach, focusing on the genetic substrates of sleep, allows for a unique exploration of how genetic predispositions might independently contribute to cognitive changes over time. This perspective is crucial because it highlights potential underlying biological mechanisms that could influence cognitive outcomes. Further, with the incorporation of brain measures, we gain a more comprehensive understanding of how brain morphometry interacts with sleep genetics and cognition. Moreover, using a comprehensive neuropsychological assessment that evaluates specific cognitive domains provides enhanced accuracy. Lastly, by building on previously reported results, this study demonstrates continuity and robustness in research findings, reinforcing the validity of the associations observed between sleep duration genetics and cognitive performance. However, there are also a few limitations that should be noted. The main limitation of this study is the relatively small sample size and the representation of only the non-Hispanic white race and ethnicity, which may reduce the generalizability of the results and limit their statistical power. Additionally, the study did not include measures to assess lifestyle changes over the 5-year period, such as variations in physical activity and diet or other factors that could significantly influence cognitive trajectories. Similarly, habitual sleep duration—a known factor affecting cognitive performance and decline—was not estimated, which may have introduced variability in the outcomes. Furthermore, the study did not account for the circadian timing of the cognitive assessments, a crucial factor as performance on cognitive tasks can fluctuate significantly depending on the time of day. Relying on a PGI based solely on sleep duration may have overlooked other critical dimensions of sleep, such as quality, timing, and consistency, which are known to influence cognitive function. This approach may also omit important genetic or behavioral factors relevant to sleep and cognition. Lastly, a potential limitation of our study is the omission of mood, particularly its relationship with sleep duration and its impact on cognitive and social cognitive functions. Mood disorders such as major depressive disorder, which are influenced by sleep patterns [49], can independently affect cognitive outcomes and may have contributed to the variability in our findings. Future analyses should account for mood as a potential confounding factor. 

In conclusion, genetic predisposition for a longer sleep duration can work as a protective factor against decline in speed of processing in young adults. Our study underscores the importance of age-specific analyses in uncovering the nuanced relationships between genetic predispositions, brain structure, and cognitive performance. 

## Figures and Tables

**Table 1:** Descriptives of our sample.

	Total	Young	Middle	Old
Age, years, mean (SD)	58.3 (15.2)	30.7 (5.9)	58.3 (5.6)	70.1 (3.8)
Sex, women, N (%)	47 (49)	8 (44.4)	20 (55.6)	19 (45.2)
Education, years, mean (SD)	16.5 (2.3)	16.4 (2.5)	16.7 (2.3)	16.5 (2.3)
Memory, mean (SD)	0.08 (0.99)	0.68 (0.96)	0.23 (0.95)	−0.31 (0.88)
Fluid reasoning, mean (SD)	0.28 (0.83)	0.86 (0.79)	0.39 (0.80)	−0.06 (0.72)
Speed of processing, mean (SD)	0.17 (0.77)	1.01 (0.76)	0.18 (0.64)	−0.19 (0.58)
Language, mean (SD)	0.32 (0.7)	0.55 (0.56)	0.48 (0.59)	0.47 (0.6)
Total, N	96	18	36	42

**Table 2:** Regression coefficients of the mixed-effect regression after being adjusted for age, sex, education, ICV, 4 PCs, time x age, and time x 4PCs (unadjusted for BMs), and those obtained after using brain measures (BM; cortical thickness and gray matter volume) as time-varying covariates (adjusted for BMs).

Cognitive Domain	Parameters	Unadjusted of BM			Adjusted for BM		
		β	95% CI	*p*	β	95% CI	*p*
Memory	Time x PGI x Age (Middle)	1.08	−21.10–23.26	0.923	0.94	−21.73–23.62	0.935
	Time x PGI x Age (Old)	5.35	−11.91–22.61	0.542	5	−12.36–22.37	0.57
Fluid Reasoning	Time x PGI x Age (Middle)	−0.74	−16.21–14.74	0.925	−2.26	−18.00–13.49	0.777
	Time x PGI x Age (Old)	0.3	−11.61–12.22	0.96	−1.61	−13.53–10.31	0.79
Speed of processing	Time x PGI x Age (Middle)	−7.84	−20.35–4.67	0.217	−9.55	−20.51–1.42	0.088
	Time x PGI x Age (Old)	−7.64	−17.22–1.94	0.117	−10.24	−18.44–−2.03	0.015
Language	Time x PGI x Age (Middle)	1.58	−8.17–11.33	0.749	3.02	−7.31–13.35	0.564
	Time x PGI x Age (Old)	2.45	−5.01–9.91	0.518	2.52	−5.25–10.28	0.523

## Data Availability

Data available upon request from the authors.

## Supplementary Materials

The following supporting information can be downloaded at: https://www.mdpi.com/article/10.3390/genes16010021/s1.
